# The Quality of Eggs Derived from Japanese Quail Fed with the Fermented and Non-Fermented Rapeseed Meal

**DOI:** 10.3390/foods11162492

**Published:** 2022-08-18

**Authors:** Karolina Wengerska, Anna Czech, Sebastian Knaga, Kamil Drabik, Tomasz Próchniak, Remigiusz Bagrowski, Angelika Gryta, Justyna Batkowska

**Affiliations:** 1 Institute of Biological Basis of Animal Production, University of Life Sciences in Lublin, 13 Akademicka St., 20-950 Lublin, Poland; 2 Department of Biochemistry and Toxicology, University of Life Sciences in Lublin, 13 Akademicka St., 20-950 Lublin, Poland; 3 Department of Physical Chemistry of Porous Materials, Institute of Agrophysics of Polish Academy of Sciences, 4 Doświadczalna St., 20-290 Lublin, Poland

**Keywords:** alternative protein source, egg quality, canola meal, fermentation

## Abstract

The most popular protein source in poultry feed mixtures is soybean. However, cheaper and more available alternative protein sources are being sought, and feed manufacturers more and more often turn their attention to the post-extraction meals of local oil plants, e.g., rapeseed. Therefore, the effect of fermented and non-fermented post-extraction rapeseed meal used as a feed additive for Japanese quails was investigated on the eggs’ quality. The study was performed on 280 females of Japanese quails fed with a mixture without rapeseed meal, with non-fermented post-extraction rapeseed meal (5%, 10% and 15%) and with fermented one (5%, 10% and 15%). During the experiment, eggs were collected from each group four times (every 4 weeks) and evaluated for their quality characteristics. The addition of 10% fermented rapeseed meal had the most beneficial effect on such eggs quality traits as egg weight, specific gravity, yolk index and color and albumen pH. However, in the majority of examined parameters, no significant differences were found between birds fed with soybean meal and those fed with fermented and non-fermented rapeseed meal (morphological elements proportions, yolk weight, albumen height and Haugh’s units, eggshell quality). This supports the thesis that the use of rapeseed meals instead of soybean meals may allow obtaining the proper quality of animal raw materials at a lower cost and with the use of local feed resources.

## 1. Introduction

Protein is one of the most important balanced components of poultry feed and the source of essential amino acids. Its quality is crucial for the proper growth and good health of birds, their production and the quality of obtained eggs [1,2]. Currently, soybean meal is a major protein source in poultry nutrition. Its impact on poultry commodities has been well known for decades. It is considered an excellent source of protein. Moreover, when properly supplemented with cereals, it fully meets the essential amino acid requirements for all livestock, making it the standard to which other plant protein sources are compared [3]. Soybean contains about 40% protein and 20% fat [4], but its nutritional composition depends mainly on the percentage of anti-nutritional factors, variety, the efficiency of the oil extraction process and the amount of husk residue or heat treatment [5]. The main problem with using soybean protein is the public’s aversion to genetically modified plants and fear of their impact on the health aspect of food products. However, the study of Ash et al. [6] showed that the laying hens are able to digest soybean meal protein to a stage where intact GMO material cannot be detected, thus demonstrating that the use of GM soybean cannot affect consumer health. Another threat to the safety of poultry raw materials and consumers is the fact that soya beams are often contaminated with microbials (*Salmonella* spp.) and need additional processing [7].

However, due to the difficulty of cultivation, the high transportation cost and the increasing consumer interest in organic products, the poultry industry is looking for a cheaper, local alternative to soybean meal sources of feed protein [8]. The locally produced oilseed meals, a by-product of oil production, are gaining increasing attention as an excellent source of protein. In the case of meat-type Japanese quails, higher than 15% rapeseed meal did not have any beneficial effect on birds’ productivity, probably due to the high content of glucosinolates. It was found that the combination of 15% soybean meal and 15% of rapeseed meal allows for achieving better weight gain, feed efficiency and dressing percentage and was economically justified [8]. In laying quails, it was noticed rapeseed meal could be used with a substituting ratio of 25% of the crude protein contributed by soybean meal regardless of additional supplementation with enzymes. The higher proportion may cause deterioration of production efficiency (growth rate, laying production); however, the quality of obtained eggs, except the yolk color, did not depend on the experimental factor [9]. Similar observations were made by Moraes et al. [10], however, using a considerably higher proportion of rapeseed meal (30%, 40% and 50% in relation to soybean meal).

The post-extraction meals may be subjected to additional treatments contributing to the increase in nutritional value, such as mentioned above enzymes additive or fermentation. The fermented meals were previously used in the feeding of many livestock with ambivalent results. For dairy cows, feeding double low rapeseed (00) resulted in increased milk protein content and milk yield, while a slight tendency toward a negative influence on the fertility of primiparous cows was noticed [11]. Fermented rapeseed meal was also used in piglets nutrition; the combination of 2% soybean meal with 6% fermented rapeseed meal improved the redox status, and a decrease in the level of markers of lipid peroxidation, LOOH and MDA in the blood was also noted [12]. However, in rabbits, the addition of fermented rapeseed meal contributed to the elongation of the cecum and large intestine, as well as lowering the pH value of the animals’ muscles while increasing their collagen content [13]. According to Wlazło et al. [14], an 8% or 12% inclusion of fermented rapeseed meal in rabbit diets resulted in a decrease in the concentration of anaerobic bacteria and *Escherichia coli* in the intestinal contents. Moreover, the addition of fermented rapeseed meals also influences a decrease in the total number of *C. perfringens* while increasing the number of mesophilic bacteria compared to conventionally fed groups in mink [15]. It was shown that fermented rapeseed meal, compared to the unfermented version, significantly improves weight gain and intestinal microflora profile and has a positive effect on meat quality and fatty acid profile, as well as increasing antioxidant activity in breast muscle. It was therefore concluded that the introduction of 50% fermented rapeseed meal in the diet of birds in place of the unfermented meal was most beneficial for broilers [16]. As shown by Zhu et al. [17], the addition of rapeseed meal to the feed of laying hens did not affect the quality of the obtained eggs, while the addition of rapeseed meal in an amount higher than 6.5 g/hen/day reduced eggs production.

The hypothesis was made that the post-extraction rapeseed meal should not deteriorate the quality of obtained Japanese quail eggs, however, the fermentation of feed material should have a more beneficial effect than non-fermented. The aim of the study was to evaluate the effect of fermented and non-fermented post-extraction rapeseed meals used as a component of feed on egg quality traits of Japanese quails.

## 2. Materials and Methods

The study was approved by the Local Ethics Committee for experiments on animals (approval no. 65/2020). The study was carried out in the laboratories of the Institute of Biological Basis of Animal Production and the Laura Kaufman Didactic and Research Station for Small Animals of the University of Life Sciences in Lublin (Poland).

A total of 280 individually wingmarked females of Japanese quail at the 5th week of age were randomly allocated into 7 experimental groups, 4 replications of 10 birds each. The schema of the experiment is presented in Table 1. Group I was the control group (C), fed a mixture without rapeseed material; groups II, III and IV were birds receiving a standard feed with rapeseed meal at 5 (RM5), 10 (RM10) and 15% (RM15) in the mixture, respectively; and groups V, VI and VII birds receiving a mixture with fermented rapeseed meal at 5 (FRM5), 10 (FRM10) and 15% (FRM15), respectively. The rapeseed meal was fermented using the strain *Bacillus subtilis* 87Y from the strain collection of InventionBio Ltd. (Bydgoszcz, Poland), prepared and analyzed as it was described before by Wlazło et al. [14].

The feed composition is shown in Table 2. Metabolizable energy was calculated according to the equation of Kirchgessner and Roth [18]. Basal nutrient contents in Feed samples were analyzed according to and for total AOAC [19] as follows: crude protein method no 954.01, crude fiber 920.39, crude fat 978.10, phosphorus was determined colorimetrically (method no 965.17), Ca was determined using the FAAS technique (method 968.08).

The study was conducted for 12 weeks. At the beginning of the experiment and every 4 weeks, 20 eggs were collected from each group, and qualitative analysis was performed using an EQM (Egg Quality Measurement, TSS^®^, York, UK) and the Instron Mini 55 apparatus (Instron^®^, Norwood, MA, USA). The following egg traits were evaluated:

1. Whole egg:
  - Weight (electronic scale with an accuracy of 0.01 g);
  - Specific gravity (measurement of the egg weight both in the air and submerged in water, according to Archimedes’ principle);
  - Proportions of morphological elements (eggshell, albumen, yolk) in the egg weight.
2. Egg yolk
  - Colour (using colorimeter according to 16-point scale, YolkFanTM, DSM Nutritional Products, Basel, Switzerland);
  - Weight (electronic balance with an accuracy of 0.01 g);
  - Index (measured using an electronic caliper, estimated as a ratio of the yolk height to its diameter);
  - pH (by ph-meter with a combined glass electrode, Elmetron^®^, Zabrze, Poland).
3. Egg albumen
  - Height (measured after the egg has been broken onto the mirror table, by contact of the EQM sensor with the surface of the dense albumen);
  - Haugh units [20];
  - Mass (from the difference in mass of whole egg, shell and yolk);
  - pH (by ph-meter with a combined glass electrode, Elmetron^®^, Zabrze, Poland).
4. Eggshell
  - Weight (electronic scale with an accuracy of 0.01 g);
  - Thickness (measured at the equator using a micrometer screw);
  - Strength (force required to break the shell (N), using an Instron Mini 55 device (Instron^®^, Norwood, MA, USA);
  - Density [21].

The data collected during the experiment were statistically processed using SPSS 24.0 package (Armonk, NY, USA) [22] using multifactorial analysis of variance with Tukey’s multiple comparisons test. The level of *p* ≤ 0.05 was taken as significant. Three factors were included in the model: time of feed administration (T), the dose of rapeseed meal (D) and fermentation of feed component (F), as well as the interactions between them (T × D, T × F, D × F, T × D × F). The exact probability of tests was given. The results were shown as the mean and standard error of the mean (SEM).

## 3. Results

The detailed means of particular traits in all evaluated groups and all terms of analyses are presented in Supplementary Tables S1–S4.

Table 3 shows the quality characteristics of whole Japanese quail eggs depending on the dose of rapeseed meal feed additive d and its type (fermented vs. non-fermented). Egg weight increased significantly with the age of the birds (Table S1), with eggs of the highest weight (10.79 g) being obtained from birds in the control group fed without the rapeseed meal. Eggs of similar weight were also obtained from all birds fed fermented post-extraction rapeseed meal. The highest egg-specific gravity (1.071 g/cm^3^) was recorded in birds from RM5 and RM15, fed with a mixture containing 5% and 15% rapeseed meal additive, respectively. There was no significant difference in the eggshell proportion with the experimental time; however, the eggs from RM15 birds had the highest shell proportion (15.62%), while the birds in the FRM15 group had the lowest one (13.80%). The highest albumen proportion was found in eggs from the FRM10 group, while the highest yolk proportion was noticed in eggs from the RM15 group. All analyzed traits of the whole egg, except the eggshell proportion, stayed under the influence of time (Table S1). The dose of the experimental factor affected the egg weight. This trait, as well as the egg-specific gravity, also depended on the rapeseed meal fermentation. The three-factorial interaction was significant only in the case of the egg weight. Egg density and albumen proportion were influenced by the single effect of feeding time and dose of rapeseed meal and its interaction.

Table 4 shows the yolk quality characteristics of Japanese quail eggs depending on the dose of rapeseed meal addition to the feed and its type. Similar to the trend stated in the case of egg weight, the yolk weight also increased with time (Table S2). During the experiment, yolk color varied in all groups, but no regular trends of this change were observed. In general, the darkest yolk color was observed in eggs from quails fed with a 10% share of rapeseed meal. There were differences between the experimental groups for the yolk index. In eggs obtained from birds in the RM5 group, the highest yolk index was stated, while in those obtained from the control group, the lowest value was significantly found, and yolks were flatter. The highest pH was observed in the RM15 group, and the lowest one was in FRM5. All yolk characteristics were affected by the age of the birds but also by the interaction of all experimental factors. Moreover, the interaction of rapeseed dose and its application time was significant in the case of all yolk traits. The acidity of the yolk was influenced by all single factors as well as all possible interactions between them.

The albumen quality characteristics of Japanese quail eggs depending on the amount of canola meal addition to the feed and its type are presented in Table 5. The significantly heaviest albumen was observed in the control and FRM10 group and its lowest weight in eggs from RM15. Eggs from RM15 were also characterized by the highest pH. The albumen height, as well as the Haugh’s units, did not change and varied considerably between groups (Table S3). The albumen pH was the only trait of this element affected by triple interaction (T × D × F). All of the albumen characteristics were changed due to the impact of time and fermentation and albumen weight, height and pH also of rapeseed meal dose. The smallest influence of factors’ interactions was found in albumen traits.

Eggshell quality traits of Japanese quail eggs depending on rapeseed meal addition to the feed and its type are presented in Table 6. There were no significant differences in any eggshell characteristics. Although their values were similar regardless of the experimental group, the impact of the analyzed factors was slightly different. All shell features stayed under the impact of all factors’ interaction, but it was the only significant influence noticed in the case of eggshell strength. The shell weight, thickness and density depended considerably on experimental time (Table S4) as well as its interactions with the dose and fermentation of rapeseed meal, respectively. The relation between dose and fermentation significantly influenced only the shell thickness.

## 4. Discussion

The addition of rapeseed meal as a source of protein in laying feed has been considered for many years [23,24], but due to its high content of glucosinolates, which cause, among others: an increase in mortality due to Fatty hemorrhagic liver syndrome, excessive carcass fat accumulation, a decrease in egg weight or a “fishy” yolk odor and the amount of the additive was limited [25,26]. It is worth noting, however, that the double zero (“00”) rapeseed was used in our study. The bacteria used in the fermentation process may carry probiotic effects. In a study by Jazi et al. [27], the digestive tract of Japanese quails fed fermented soybean meal contained more lactic acid bacteria with a lower total bacterial count compared to birds fed with soybean meal. According to Szmigiel et al. [28], the histological structure of the cecum of broilers given fermented rapeseed meal was improved. Interestingly, the authors showed a similar effect with unfermented rapeseed meals. Similar conclusions were reached by Elbaz [29], who observed the best intestinal health in birds fed with a fermented rapeseed meal compared to an unfermented one as well mix of rapeseed meal with probiotic and feed mixture without rapeseed. It is noteworthy that not all fermented protein alternatives carry positive effects as demonstrated by Choi et al. [30] fermented seaweed fusiforme decreased Haugh’s units compared to fermented brown seaweed.

In contrast to our study, Kiiskinen [31] and Olomu et al. [32] observed no change in egg weight depending on the addition of various levels of rapeseed meal. One of the differences in egg weight registered could also be the fermentation of rapeseed meal used in our study due to this process contributes to the decrease in anti-nutritional compounds in the feed mixture material [33]. The lack of significant change in eggshell percentage in our study is contrary to the study of Saki et al. [34], who observed a significant reduction in shell proportion depending on the increasing levels of rapeseed meal in quail diets. Similar conclusions were reached by Kaminska [35], in whose study increasing rapeseed meal dose led to a reduction in eggshell proportion in both, Hy-Line and ISA Brown hens. In the present study, there was no statistical difference in albumen proportion between groups; however, the highest value of this trait was recorded in eggs from the control and FRM10 birds. As shown in the study by Shi et al. [36], the addition of about 16% sunflower meal has the most beneficial effect on the albumen content in eggs compared to the control group and birds fed with 8% and 25% sunflower meal.

Considerably more intense yolk color was found in eggs from RM10 and RM15 groups in comparison to other groups of quails. This yolk’s color corresponded to 10 points on the Roche scale. More intensive coloration is advantageous from the producer’s point of view since it was shown that eggs with darker yolk colors are more readily chosen by consumers [37]. Our results are in line with those obtained by Moraes et al. [10]; the increase in canola meal dose contributed to better (more intensive) yolk coloration. Moreover, in a study by Blair et al. [26], the addition of rapeseed meals fed to Babcock hens tended to darken yolk. On the other hand, Świątkiewicz et al. [38] observed slightly less colored egg yolks (about four points on the Roche scale) when Japanese quails were fed with rapeseed cake. The positive change in yolk color could also result from slightly higher crude fiber content related to the amount of rapeseed meal. This characteristic is one of the few egg quality traits affected by varying amounts of fiber in the feed [39].

In our study, the best albumen quality was characterized by eggs from the RM5 group (88.00 HU). These results are consistent with those obtained by Thomas et al. [40], who showed that quails fed with the addition of 100, 125 and 150 g/kg low glucosinolate rapeseed meal had the best albumen quality expressed in Haugh’s units than the addition of 100 and 150 g/kg high-glucosinolate rapeseed meal. Similar conclusions were reached by Richter et al. [41], who analyzed the quality of hen eggs depending on the level of low-glucosinolate rapeseed. Disparate results on albumen quality were presented by Gheisari and Ghayor [42], in which eggs obtained from quail fed a mixture containing 15% canola meal were characterized by a significantly better quality of this morphological element, while the best albumen quality characterized eggs from birds fed with 20% canola meal addition. A slightly different observation was made by Wang et al. [2], who analyzed the quality of eggs from hens fed various alternative protein sources (including rapeseed meals). It was found that eggs from birds fed with rapeseed meals were characterized by lower quality compared to those fed with a standard feed mixture (based on soybean meal).

Although no statistical differences were found in the most important eggshell characteristics, it was seen that eggshells from groups fed with both non-fermented and fermented rapeseed meals had better quality. Therefore, rapeseed meals, especially fermented ones, probably contributed to higher calcium retention [38], preventing unfavorable changes in shell structure. This hypothesis seems to be confirmed by our research. The results regarding the change in shell thickness are only partially in agreement with the study of Saki et al. [16], in which Japanese quails from the control group were characterized by the thicker shell than groups fed with rapeseed meal. Similarly, Tan et al. [27] found that dietary inclusion of 10% rapeseed meal decreased egg weight and eggshell thickness of duck eggs. In our study, no differences in eggshell quality traits were shown; however, the highest shell strength was found in eggs from the RM10 group. These results are similar to Wang et al. [27], who compared soybean meal, low-gossypol cottonseed meal and double-zero rapeseed meal and showed no differences in eggshell quality traits. On the other hand, as the study by Kopacz et al., (2020) pointed out that the treatment of rapeseed meal (i.e., fermentation) included in the birds’ diet can affect the changes in eggshell strength; however, the results obtained in cited work for raw and fermented raw material did not show significant differences in this regard, as in our study.

## 5. Conclusions

In most examined parameters of egg quality traits, no significant differences could be found between birds fed with soybean meal and birds fed with fermented and non-fermented rapeseed meal. This confirms the thesis that the use of rapeseed meals instead of traditional soybean meals may allow obtaining the correct quality of animal raw materials, but at a lower cost and with the use of local feed resources.

## Figures and Tables

**Table 1:** The schema of the experiment.

Group	I	II	III	IV	V	VI	VII
	C	RM5	RM10	RM15	FRM5	FRM10	FRM15
Feeding regime							
Rapeseed meal (%)	0	5	10	15			
Fermented rapeseed meal (%)	0				5	10	15
Birds							
No. of replications	4	4	4	4	4	4	4
No. of birds	40	40	40	40	40	40	40
Eggs							
Time (wks)	No. of evaluated eggs						
0	20	20	20	20	20	20	20
4	20	20	20	20	20	20	20
8	20	20	20	20	20	20	20
12	20	20	20	20	20	20	20
Total	80	80	80	80	80	80	80

**Table 2:** The composition if feed mixture used in the experiment.

Ingredients (%)	Group						
	I	II	III	IV	V	VI	VII
	C	RM5	RM10	RM15	FRM5	FRM10	FRM15
Wheat	16.59	23.03	27.21	25.01	23.76	28.36	26.74
Triticale	10.00	10.00	10.00	10.00	10.00	10.00	10.00
Corn	15.00	15.00	15.00	15.00	15.00	15.00	15.00
Soybean meal	30.31	27.40	24.46	21.43	26.98	23.62	20.17
Wheat bran	13.13	5.58			5.45		
Rapeseed meal		5.00	10.00	15.00			
Fermented rapeseed meal					5.00	10.00	15.00
Soybean oil	5.87	4.92	4.30	4.62	4.75	4.00	4.18
Salt	0.36	0.34	0.32	0.30	0.34	0.32	0.30
Limestone	6.32	6.24	6.19	6.18	6.27	6.24	6.25
1-Calcium phosphate	1.77	1.80	1.80	1.70	1.75	1.70	1.56
Methionine 99 DL	0.09	0.07	0.06	0.05	0.07	0.06	0.05
L-Lysine	0.07	0.12	0.17	0.21	0.14	0.20	0.25
Vitamin-mineral premix ^1^	0.50	0.50	0.50	0.50	0.50	0.50	0.50
Total	100.00	100.00	100.00	100.00	100.00	100.00	100.00
Metabolic energy (kcal) ^#^	2800	2800	2800	2800	2800	2800	2800
Crude protein (%)	21.00	21.00	21.00	21.00	21.00	21.00	21.00
Crude fiber (%)	3.50	3.50	3.64	4.17	3.50	3.66	4.20
Lysine (%) ^#^	1.15	1.15	1.15	1.15	1.15	1.15	1.15
Methionine (%) ^#^	0.39	0.39	0.39	0.39	0.39	0.39	0.39
Met + Cys (%) ^#^	0.75	0.75	0.75	0.75	0.75	0.75	0.75
Threonine (%) ^#^	0.78	0.79	0.80	0.81	0.77	0.76	0.75
Ca (%)	2.90	2.90	2.90	2.90	2.90	2.90	2.90
P(%)	0.85	0.84	0.83	0.83	0.83	0.86	0.84

^#^ Calculated parameters, ^1^ composition of the mineral–vitamin premix: vitamin A—2,400,000 IU, vitamin D3—600,000 IU, vitamin E—0.0 mg, vitamin K—1000 mg, vitamin B1—600 mg, vitamin B2—2400 mg, vitamin B6—1000 mg, vitamin B12—6 mg, folic acid—400 mg, biotin—60 mg, nicotinic acid—10,000 mg, calcium pantothenate—2600 mg, Mn—24 g, Zn—20 g, Fe—10 g, Cu—2 g, J—400 mg, Se—0 mg, Co—30 mg, phytase—750 FTU; C—control group; RM5, RM10, RM15—5%, 10%, 15% of post-extraction rapeseed meal, respectively; FRM5, FRM10, FRM15—5%, 10%, 15% of fermented post-extraction rapeseed meal, respectively.

**Table 3:** The quality characteristics of whole eggs of Japanese quails depend on the dose and fermentation of rapeseed meal applied.

Trait		Weight (g)	Specific Gravity (g/cm^3^)	Proportions (%)		
				Yolk	Albumen	Eggshell
Group	C	10.79 ^b^	1.068 ^ab^	29.36	57.34	13.92
	RM5	10.42 ^ab^	1.071 ^b^	29.47	56.94	13.93
	RM10	10.57 ^ab^	1.069 ^ab^	29.39	57.55	14.02
	RM15	10.32 ^a^	1.071 ^ab^	31.59	56.76	15.62
	FRM5	10.57 ^ab^	1.068 ^a^	29.59	57.58	13.97
	FRM10	10.75 ^b^	1.069 ^ab^	28.73	57.72	13.93
	FRM15	10.63 ^ab^	1.070 ^ab^	29.59	56.62	13.80
	SEM	0.035	0.000	0.411	0.166	0.239
Factor (*p-value*)	T	*0.000*	*0.000*	*0.001*	*0.000*	*0.716*
	D	*0.001*	*0.256*	*0.385*	*0.118*	*0.485*
	F	*0.008*	*0.011*	*0.391*	*0.474*	*0.188*
	T × D	*0.275*	*0.037*	*0.082*	*0.000*	*0.127*
	T × F	*0.317*	*0.002*	*0.124*	*0.020*	*0.057*
	D × F	*0.431*	*0.239*	*0.681*	*0.386*	*0.247*
	T × D × F	*0.009*	*0.147*	*0.395*	*0.016*	*0.022*

^a, b^—means in the same column are significant at *p* < 0.05; SEM—standard error of mean; C—control group; RM5, RM10, RM15—5%, 10%, 15% of post-extraction rapeseed meal, respectively; FRM5, FRM10, FRM15—5%, 10%, 15% of fermented post-extraction rapeseed meal, respectively.

**Table 4:** The quality characteristics of yolk of Japanese quails eggs depending on the dose and fermentation of rapeseed meal applied.

Trait		Weight (g)	Colour (pts)	Index	Acidity (pH)
Group	C	3.09	9.43 ^a^	0.399 ^a^	6.05 ^a^
	RM5	3.10	9.62 ^ab^	0.424 ^c^	6.11 ^ab^
	RM10	3.01	10.12 ^c^	0.410 ^abc^	6.12 ^ab^
	RM15	3.13	10.00 ^c^	0.411 ^abc^	6.18 ^b^
	FRM5	3.08	9.90 ^bc^	0.402 ^ab^	6.02 ^a^
	FRM10	3.14	9.96 ^bc^	0.414 ^abc^	6.08 ^ab^
	FRM15	3.14	9.92 ^bc^	0.420 ^ba^	6.10 ^ab^
	SEM	0.017	0.034	0.002	0.010
Factor (*p-value*)	T	*0.000*	*0.000*	*0.000*	*0.000*
	D	*0.405*	*0.000*	*0.145*	*0.000*
	F	*0.171*	*0.478*	*0.633*	*0.000*
	T × D	*0.000*	*0.000*	*0.000*	*0.000*
	T × F	*0.042*	*0.035*	*0.442*	*0.000*
	D × F	*0.065*	*0.144*	*0.013*	*0.003*
	T × D × F	*0.000*	*0.000*	*0.013*	*0.000*

^a–c^—means in the same column are significant at *p* < 0.05; SEM—standard error of mean; C—control group; RM5, RM10, RM15—5%, 10%, 15% of post-extraction rapeseed meal, respectively; FRM5, FRM10, FRM15—5%, 10%, 15% of fermented post-extraction rapeseed meal, respectively.

**Table 5:** The albumen quality characteristics of Japanese quail eggs depending on the dose and fermentation of rapeseed meal applied.

Trait		Weight (g)	Height (mm)	Haugh Units	Alkalinity (pH)
Group	C	6.19 ^b^	4.01	86.97	8.94 ^ab^
	RM5	5.94 ^ab^	4.11	88.00	9.03 ^b^
	RM10	6.08 ^ab^	4.07	87.64	8.99 ^ab^
	RM15	5.89 ^a^	3.82	86.61	9.03 ^b^
	FRM5	6.09 ^ab^	3.94	86.75	9.02 ^b^
	FRM10	6.20 ^b^	3.92	86.54	8.82 ^a^
	FRM15	6.01 ^ab^	3.84	86.19	8.91 ^ab^
	SEM	0.026	0.029	0.172	0.017
Factor (*p-value*)	T	*0.000*	*0.000*	*0.000*	*0.000*
	D	*0.003*	*0.040*	*0.530*	*0.000*
	F	*0.047*	*0.017*	*0.002*	*0.000*
	T × D	*0.011*	*0.002*	*0.001*	*0.000*
	T × F	*0.235*	*0.011*	*0.006*	*0.001*
	D × F	*0.737*	*0.595*	*0.705*	*0.164*
	T × D × F	*0.098*	*0.325*	*0.121*	*0.000*

^a, b^—means in the same column are significant at *p* < 0.05; SEM—standard error of mean; C—control group; RM5, RM10, RM15—5%, 10%, 15% of post-extraction rapeseed meal, respectively; FRM5, FRM10, FRM15—5%, 10%, 15% of fermented post-extraction rapeseed meal, respectively.

**Table 6:** Eggshell quality traits of Japanese quail eggs depending on the dose and fermentation of rapeseed meal applied.

Trait		Strength (N)	Weight (g)	Thickness (mm)	Density (g/cm^3^)
Group	C	11.81	1.50	0.182	3.65
	RM5	11.79	1.44	0.187	3.45
	RM10	12.59	1.48	0.184	3.54
	RM15	12.20	1.47	0.188	3.66
	FRM5	12.23	1.47	0.191	3.44
	FRM10	12.07	1.49	0.191	3.42
	FRM15	12.49	1.47	0.183	3.56
	SEM	0.111	0.007	0.001	0.028
Factor (*p-value*)	T	*0.193*	*0.000*	*0.000*	*0.000*
	D	*0.416*	*0.186*	*0.185*	*0.613*
	F	*0.944*	*0.968*	*0.802*	*0.458*
	T × D	*0.222*	*0.000*	*0.000*	*0.000*
	T × F	*0.053*	*0.000*	*0.000*	*0.000*
	D × F	*0.163*	*0.656*	*0.006*	*0.309*
	T × D × F	*0.027*	*0.000*	*0.001*	*0.000*

SEM—standard error of mean; C—control group; RM5, RM10, RM15—5%, 10%, 15% of post-extraction rapeseed meal, respectively; FRM5, FRM10, FRM15—5%, 10%, 15% of fermented post-extraction rapeseed meal, respectively.

## Data Availability

The data presented in this study are available on request from the corresponding author.

## Supplementary Materials

The following supporting information can be downloaded at: https://www.mdpi.com/article/10.3390/foods11162492/s1, Table S1: The quality characteristics of whole eggs of Japanese quails depending on the dose and fermentation of rapeseed meal applied; Table S2: The quality characteristics of yolk of Japanese quails eggs depending on the dose and fermentation of rapeseed meal applied; Table S3: The albumen quality characteristics of Japanese quail eggs depending on the dose and fermentation of rapeseed meal applied; Table S4: Eggshell quality traits of Japanese quail eggs depending on the dose and fermentation of rapeseed meal applied.
