# Supplementary material for: The Quality of Eggs Derived from Japanese Quail Fed with the Fermented and Non-Fermented Rapeseed Meal
Source: Foods. 2022 Aug 18;11(16):2492. doi: 10.3390/foods11162492 (PMC9407498; doi:10.3390/foods11162492)
Supplement: Supplementary file 1 [file foods-11-02492-s001.zip › foods-1861438-supplementary.pdf]

Table S1.The quality characteristics of whole eggs of Japanese quails depending on the dose and fermentation of rapeseed meal applied

Group			C	RM5	RM10	RM15	FRM5	FRM10	FRM15	Total	SEM
Trait		term (wks)									
Egg weight (g)		0	9.72_{w}	10.1_{w}	9.82_{w}	9.35_{w}	9.48_{w}	9.92_{w}	9.94_{w}	9.97_{w}	*0.101*
		4	10.38^{bc}_{w}	9.81^{a}_{w}	10.01^{abc}_{w}	10.11^{abc}_{x}	10.09^{abc}_{x}	10.53^{c}_{x}	9.93^{ab}_{w}	10.12_{x}	*0.073*
		8	11.25^{b}_{x}	10.70^{ab}_{x}	10.83^{ab}_{x}	11.04^{a}_{xy}	11.14^{b}_{y}	10.99^{ab}_{xy}	11.09^{b}_{x}	10.86_{y}	*0.128*
		12	11.54^{b}_{x}	10.95^{ab}_{x}	11.28^{ab}_{x}	11.12^{ab}_{y}	11.08^{ab}_{y}	11.30^{ab}_{y}	11.52^{b}_{x}	11.25_{z}	*0.066*
		Total	10.79^{b}	10.42^{ab}	10.57^{b}	10.14^{a}	10.57^{b}	10.75^{b}	10.63^{b}		*0.054*
Specific gravity		0	1.072_{x}	1.077_{y}	1.076_{x}	1.075_{y}	1.074_{x}	1.074	1.074_{y}	1.075_{y}	*0.0007*
		4	1.069^{ab}_{wx}	1.069^{ab}_{wx}	1.067^{a}_{w}	1.071^{ab}_{x}	1.070^{ab}_{x}	1.068^{ab}	1.073^{b}_{xy}	1.070_{x}	*0.0007*
		8	1.065^{ab}_{w}	1.073^{b}_{xy}	1.068^{ab}_{w}	1.069^{ab}_{wx}	1.062^{a}_{w}	1.068^{ab}	1.064^{ab}_{w}	1.067_{w}	*0.0012*
		12	1.069_{wx}	1.068_{w}	1.068_{w}	1.067_{x}	1.067_{wx}	1.068	1.068_{wx}	1.070_{x}	*0.0005*
		Total	1.068^{ab}	1.071^{b}	1.069^{ab}	1.070^{ab}	1.067^{a}	1.069^{ab}	1.070^{ab}		*0.000*
Proportions (%)	eggshell	0	12.80^{a}_{w}	14.50^{b}_{x}	15.72^{b}_{x}	14.34^{b}	14.87^{b}_{x}	14.26^{ab}_{wx}	15.17^{b}_{y}	14.37	*0.201*
		4	14.2^{ab}_{x}	14.49^{b}_{x}	13.43^{ab}_{y}	13.01^{a}	13.71^{ab}_{x}	13.67^{ab}_{wx}	13.79^{ab}_{x}	13.77	*0.170*
		8	14.51^{a}_{x}	13.19^{a}_{w}	13.89^{a}_{y}	15.61^{b}	13.47^{a}_{x}	13.40^{a}_{w}	12.84^{a}_{w}	14.67	*1.835*
		12	13.78_{wx}	13.72_{wx}	13.88_{y}	13.78	14.24_{wx}	14.51_{x}	13.96_{xy}	13.98	*0.126*
		Total	13.91^{a}	13.93^{a}	14.02^{a}	18.20^{b}	13.97^{a}	13.93^{a}	13.92^{a}		*0.499*
	albumen	0	61.39^{c}_{y}	57.58a	57.54^{a}	59.49^{abc}_{y}	59.88^{abc}_{x}	60.57^{bc}_{y}	58.37^{bc}_{y}	59.32_{x}	*0.384*
		4	57.10_{wx}	57.12	58.18	58.72_{y}	57.59_{wx}	57.82_{x}	57.37_{xy}	57.70_{wy}	*0.219*
		8	58.45^{b}_{xy}	57.86^{b}	58.57^{b}	53.00^{a}_{w}	60.15_{bx}	58.22^{b}_{x}	55.84^{b}_{wx}	54.71_{w}	*2.821*
		12	53.78_{w}	55.44	55.80	55.66_{x}	53.72_{w}	55.13_{w}	54.96_{w}	$54{,}92_{w}$	*0.281*
		Total	57.45	56.94	57.55	52.00	57.58	57.72	56.62		*0.773*
	yolk	0	25.81^{ab}_{w}	27.92^{b}_{w}	26.73^{ab}_{w}	26.18^{ab}	25.25^{a}_{w}	26.46^{ab}_{w}	26.45^{ab}_{w}	26.39_{w}	*0.320*
		4	28.63_{w}	28.40_{w}	28.39_{x}	28.27	28.69_{x}	28.51_{w}	28.84_{x}	28.53_{w}	*0.157*
		8	29.18_{w}	30.20_{x}	30.83_{y}	31.08	29.91_{xy}	28.39_{w}	31.32_{y}	35.15_{z}	*3.618*
		12	32.44^{b}_{x}	30.84^{ab}_{x}	30.32^{a}_{y}	30.56^{a}	30.03^{ab}_{y}	30.36^{a}_{x}	31.00^{ab}_{y}	31.10_{wx}	*0.229*
		Total	29.23^{a}	29.47^{a}	29.39^{a}	37.74^{b}	29.44^{a}	28.59^{a}	29.46^{a}		*0.988*

$^{a-d}$– means in the same row (group) significant at p≤0.05; $_{w-z}$ - means in the same column (terms) significant at p≤0.05; SEM+ standard error of mean; C - control group; RM5, RM10, RM15– 5%, 10%, 15% of post-extraction rapeseed meal respectively; FRM5, FRM10, FRM15– 5%, 10%, 15% of fermented post-extraction rapeseed meal respectively

Table S2. The quality characteristics of yolk of Japanese quails eggs depending on the dose and fermentation of rapeseed meal applied

Group		C	RM5	RM10	RM15	FRM5	FRM10	FRM15	Total	SEM
Trait	term (wks)									
Colour (pts)	0	9.80^{b}_{x}	9.46^{ab}_{wx}	8.80^{a}_{w}	8.66^{a}_{w}	9.27^{ab}_{w}	9.07^{ab}_{w}	8.86^{a}_{w}	9.07_{w}	*0.122*
	4	10.05^{a}_{x}	10.50^{bc}_{y}	10.80^{c}_{y}	10.80^{c}_{y}	10.60^{bc}_{x}	10.70^{bc}_{y}	10.30^{ab}_{x}	10.54_{z}	*0.055*
	8	8.95^{a}_{w}	9.55^{ab}_{x}	$10.05b^{c}_{x}$	10.45^{c}_{y}	10.25^{c}_{x}	10.00^{bc}_{x}	10.45^{c}_{x}	9.95_{y}	*0.091*
	12	9.05^{ab}_{w}	8.90^{a}_{w}	$10,16^{d}_{x}$	9.65^{bcd}_{x}	9.20^{abc}_{w}	9.80^{cd}_{x}	10.20^{d}_{x}	9.56_{x}	*0.103*
	Total	9.43^{a}	9.62^{ab}	10.12^{c}	9.88^{bc}	9.90^{bc}	9.96^{bc}	9.92^{bc}		*0.051*
Weight (g)	0	2.49^{a}_{w}	2.85^{b}_{w}	2.62^{ab}_{w}	2.44^{a}_{w}	2.40^{a}_{w}	2.61^{ab}_{w}	2.62^{ab}_{w}	2.57_{w}	*0.039*
	4	2.98^{ab}_{x}	2.78^{a}_{w}	2.83^{ab}_{x}	2.86^{ab}_{x}	2.90^{ab}_{x}	3.00^{ab}_{x}	2.83^{ab}_{w}	2.89_{x}	*0.027*
	8	3.26^{a}_{x}	3.27^{ab}_{x}	3.34^{bc}_{y}	3.42^{c}_{y}	3.35^{c}_{y}	3.11^{bc}_{x}	3.48^{c}_{x}	3.32_{y}	*0.047*
	12	3.75^{ab}_{y}	3.38^{a}_{x}	3.42^{d}_{y}	3.40^{bcd}_{y}	3.54^{abc}_{y}	3.44^{cd}_{y}	3.58^{d}_{x}	3.50_{z}	*0.031*
	Total	3.16	3.09	3.11	3.02	3.13	3.08	3.14		*0.024*
Acidity (pH)	0	6.03_{w}	6.10_{w}	6.15_{x}	6.36_{y}	5.98_{x}	6.31	6.25_{x}	6.17_{x}	*0.030*
	4	6.23^{a}_{y}	6.28^{ab}_{y}	6.37^{c}_{y}	6.38^{c}_{z}	6.32^{c}_{y}	6.32^{bc}	6.33^{abc}_{y}	6.31_{y}	*0.009*
	8	6.10^{bc}_{z}	6.21^{c}_{x}	6.09^{c}_{w}	6.26^{bc}_{x}	5.98^{bc}_{x}	6.18^{a}	6.20^{a}_{w}	6.14_{x}	*0.010*
	12	5.83^{bc}_{x}	5.85^{c}_{w}	5.84^{c}_{w}	5.84^{bc}_{w}	5.79^{bc}_{w}	5.69^{a}	5.77^{b}_{x}	5.80_{w}	*0.009*
	Total	6.05^{ab}	6.11^{ab}	6.11^{ab}	6.18^{b}	6.02^{a}	6.08^{ab}	6.09^{ab}		*0.014*

$^{a-d}$– means in the same row (group) significant at $p \leq 0.05$; $_{w-z}$ - means in the same column (terms) significant at $p \leq 0.05$; SEM+ standard error of mean; C - control group; RM5, RM10, RM15– 5%, 10%, 15% of post-extraction rapeseed meal respectively; FRM5, FRM10, FRM15– 5%, 10%, 15% of fermented post-extraction rapeseed meal respectively

Table S3. The albumen quality characteristics of Japanese quail eggs depending on the dose and fermentation of rapeseed meal applied

Group		C	RM5	RM10	RM15	FRM5	FRM10	FRM15	Total	SEM
Trait	term (wks)									
Height (mm)	0	3.78^{d}_{w}	3.58^{bcd}_{w}	3.66^{cd}_{w}	3.09^{abc}_{w}	3.61^{cd}_{w}	2.94^{ab}_{w}	2.82^{a}_{w}	3.30_{w}	*0.088*
	4	4.04_{w}	4.06_{w}	3.77_{w}	3.95_{x}	4.02_{w}	4.01_{x}	4.06_{z}	3.99_{x}	*0.057*
	8	3.63_{w}	3.89_{w}	3.87_{w}	3.86_{x}	3.45_{w}	3.76_{x}	3.86_{z}	3.76_{y}	*0.075*
	12	4.54_{x}	4.72_{x}	4.81_{x}	4.40_{y}	4.53_{x}	4.66_{y}	4.54_{y}	4.60_{z}	*0.070*
	Total	4.01	4.11	4.07	3.82	3.94	3.92	3.84		*0.040*
Haugh'sunits	0	86.71^{c}_{w}	85.05^{bc}_{w}	85.96^{c}_{w}	82.87^{abc}_{w}	85.77^{c}_{wx}	81.29^{ab}_{w}	80.51^{a}_{w}	83.67_{w}	*0.546*
	4	87.12_{wx}	88.34_{xy}	86.42_{w}	87.42_{x}	87.98_{xy}	87.50_{xy}	88.32_{xy}	87.60_{x}	*0.319*
	8	84.35^{ab}_{w}	86.56^{ab}_{wx}	86.35^{ab}_{w}	87.13^{b}_{x}	83.08^{a}_{w}	85.43^{ab}_{x}	86.04^{ab}_{x}	85.55_{y}	*0.513*
	12	89.69_{x}	91.02_{y}	91.18_{y}	89.22_{x}	89.71_{y}	90.35_{y}	89.59_{y}	90.10_{z}	*0.400*
	Total	86.97	88.00	87.64	86.61	86.74	86.53	86.18		*0.244*
Weight (g)	0	5.99	5.82_{w}	5.66_{w}	5.57_{w}	5.67_{w}	6.02	5.82_{wx}	5.73_{w}	*0.078*
	4	5.92^{ab}	5.61^{a}_{w}	5.82^{ab}_{wx}	5.94^{ab}_{wx}	5.81^{ab}_{w}	6.09^{b}	5.69^{a}_{w}	5.84_{w}	*0.050*
	8	6.65^{b}	6.20^{ab}_{x}	6.33^{ab}_{y}	5.39^{a}_{wx}	6.70^{b}_{x}	6.40^{b}	6.18^{ab}_{xy}	6.26_{x}	*0.123*
	12	6.21	6.08_{x}	6.29_{xy}	6.20_{x}	5.98_{w}	6.23	6.34_{y}	6.19_{x}	*0.051*
	Total	6.21^{b}	5.93^{ab}	6.08^{ab}	5.77^{a}	6.08^{ab}	6.19^{b}	6.01^{ab}		*0.044*
Alkalinity (pH)	0	8.40_{x}	8.90_{x}	9.00_{y}	9.07_{y}	9.10_{x}	8.93_{y}	8.86_{y}	8.89_{w}	*0.049*
	4	9.24^{ab}_{y}	9.27^{b}_{z}	9.33^{b}_{z}	9.24^{b}_{y}	9.20^{ab}_{y}	8.55^{a}_{y}	9.29^{b}_{z}	9.16_{x}	*0.065*
	8	9.00^{a}_{x}	9.01^{b}_{y}	8.83^{b}_{x}	9.05^{b}_{x}	9.05^{ab}_{x}	9.12^{ab}_{x}	8.63^{b}_{x}	8.95_{w}	*0.052*
	12	8.71^{a}_{w}	8.85^{b}_{w}	8.82^{b}_{w}	8.82^{b}_{w}	8.78^{ab}_{w}	8.78^{ab}_{w}	8.84^{b}_{w}	8.80_{w}	*0.009*
	Total	8.94	9.03	8.99	9.03	9.02	8.82	8.91		*0.024*

$^{a-d}$– means in the same row (group) significant at p≤0.05; $_{w-z}$ - means in the same column (terms) significant at p≤0.05; SEM+ standard error of mean; C - control group; RM5, RM10, RM15– 5%, 10%, 15% of post-extraction rapeseed meal respectively; FRM5, FRM10, FRM15– 5%, 10%, 15% of fermented post-extraction rapeseed meal respectively

Table S4. Eggshell quality traits of Japanese quail eggs depending on the dose and fermentation of rapeseed meal applied

Group		C	RM5	RM10	RM15	FRM5	FRM10	FRM15	Total	SEM
Trait	term (wks)									
Weight (g)	0	1.25$^{a}_{w}$	1.46^{bc}	1.54$^{c}_{x}$	1.34$^{ab}_{w}$	1.41$^{abc}_{w}$	1.40$^{abc}_{w}$	1.50$^{bc}_{xy}$	1.41$_{w}$	*0.022*
	4	1.48$^{b}_{x}$	1.42^{ab}	1.35$^{ab}_{w}$	1.32$^{a}_{w}$	1.37$^{ab}_{w}$	1.43$^{ab}_{w}$	1.37$^{ab}_{w}$	1.39$_{w}$	*0.018*
	8	1.62$^{bc}_{x}$	1.41^{a}	1.51$^{ab}_{w}$	1.69$^{c}_{y}$	1.50$^{ab}_{x}$	1.47$^{a}_{w}$	1.42$^{a}_{w}$x	1.52$_{x}$	*0.019*
	12	1.59$^{ab}_{x}$	1.50^{a}	1.57$^{ab}_{x}$	1.53$^{a}_{x}$	1.57$^{ab}_{x}$	1.64$^{b}_{x}$	1.61$^{ab}_{y}$	1.57$_{y}$	*0.015*
	Total	1.50	1.44	1.48	1.46	1.47	1.49	1.47		*0.010*
Thickness (mm)	0	0.196c	0.184^{bc}	0.169^{ab}	0.168^{ab}	0.153^{a}	0.164^{ab}	0.163^{ab}	0.171$_{w}$	*0.003*
	4	0.171^{a}	0.173^{a}	0.179^{ab}	0.185^{b}	0.184^{b}	0.183^{b}	0.180^{ab}	0.179$_{x}$	*0.001*
	8	0.177^{a}	0.198^{bc}	0.192^{b}	0.212^{cd}	0.219^{d}	0.218^{d}	0.204^{bcd}	0.203$_{z}$	*0.002*
	12	0.187	0.191	0.189	0.188	0.193	0.191	0.182	0.186$_{y}$	*0.003*
	Total	0.182	0.187	0.184	0.188	0.191	0.191	0.182		*0.001*
Strength (N)	0	10.66	10.20	12.99	11.94	11.38	10.95	12.13	11.53	*0.356*
	4	12.55	10.80	11.51	13.01	12.01	12.85	12.15	12.13	*0.305*
	8	10.31^{a}	12.88^{ab}	13.38^{b}	11.87^{ab}	11.57^{ab}	11.49^{ab}	12.15^{ab}	11.93	*0.340*
	12	12.47	11.39	12.01	11.82	12.45	11.90	12.04	12.01	*0.341*
	Total	11.54	11.43	12.40	12.15	11.89	11.86	12.12		*0.168*
Density (g/cm^{3})	0	0.309$^{a}_{w}$	0.356$^{ab}_{x}$	0.420$^{c}_{x}$	0.388^{bc}	0.440$^{c}_{y}$	0.417$^{bc}_{y}$	0.423$^{c}_{y}$	0.391$_{x}$	*0.009*
	4	0.384$^{c}_{y}$	0.382$^{bc}_{x}$	0.341$^{ab}_{w}$	0.319^{a}	0.339$^{a}_{x}$	0.342$^{abc}_{wx}$	0.343$^{abc}_{x}$	0.350$_{w}$	*0.006*
	8	0.385$^{ab}_{y}$	0.308$^{a}_{w}$	0.334$^{a}_{w}$	0.469^{b}	0.285$^{a}_{w}$	0.287$^{a}_{w}$	0.295$^{a}_{w}$	0.338$_{w}$	*0.015*
	12	0.365$_{xy}$	0.339$_{x}$	0.351$_{w}$	0.350	0.346$_{x}$	0.362$_{xy}$	0.369$_{x}$	0.354$_{w}$	*0.005*
	Total	0.365^{a}	0.345^{ab}	0.354^{ab}	0.382^{b}	0.312^{ab}	0.347^{ab}	0.356^{ab}		*0.005*

$^{a-d}$– means in the same row (group) significant at p≤0.05; $_{w-z}$ - means in the same column (terms) significant at p≤0.05; SEM+ standard error of mean; C - control group; RM5, RM10, RM15– 5%, 10%, 15% of post-extraction rapeseed meal respectively; FRM5, FRM10, FRM15– 5%, 10%, 15% of fermented post-extraction rapeseed meal respectively
